# Validation and analysis of expression, prognosis and immune infiltration of WNT gene family in non-small cell lung cancer

**DOI:** 10.3389/fonc.2022.911316

**Published:** 2022-07-25

**Authors:** Jianglin Wang, Qingping Yang, Mengjie Tang, Wei Liu

**Affiliations:** ^1^ Department of Pharmacy, The Third Xiangya Hospital, Central South University, Changsha, China; ^2^ Department of Pharmacy, Zunyi Medical University, Zunyi, China; ^3^ Department of Pathology, Hunan Cancer Hospital, The Affiliated Cancer Hospital of Xiangya School of Medicine, Central South University, Changsha, China

**Keywords:** NSCLC, LUAD, LUSC, WNT gene family, prognosis, immune infiltration

## Abstract

Early diagnosis and prognosis prediction of non-small cell lung cancer (NSCLC) have been challenging. Signaling cascades involving the Wingless-type (WNT) gene family play important biological roles and show prognostic value in various cancers, including NSCLC. On this basis, this study aimed to investigate the significance of WNTs in the prognosis and tumor immunity in NSCLC by comprehensive analysis. Expression and methylation levels of WNTs were obtained from the ONCOMINE, TIMER, and UALCAN. The dataset obtained from The Cancer Genome Atlas (TCGA) was utilized for prognostic analysis. cBioPortal was used to perform genetic alterations and correlation analysis of WNTs. R software was employed for functional enrichment and pathway analysis, partial statistics, and graph drawing. TRRUST was used to find key transcription factors. GEPIA was utilized for the analysis of expression, pathological staging, etc. Correlative analysis of immune infiltrates from TIMER. TISIDB was used for further immune infiltration validation analysis. Compared with that of normal tissues, WNT2/2B/3A/4/7A/9A/9B/11 expressions decreased, while WNT3/5B/6/7B/8B/10A/10B/16 expressions increased in lung adenocarcinoma (LUAD); WNT2/3A/7A/11 expressions were lessened, while WNT2B/3/5A/5B/6/7B/10A/10B/16 expressions were enhanced in squamous cell lung cancer (LUSC). Survival analysis revealed that highly expressed WNT2B and lowly expressed WNT7A predicted better prognostic outcomes in LUAD and LUSC. In the study of immune infiltration levels, WNT2, WNT9B, and WNT10A were positively correlated with six immune cells in LUAD; WNT1, WNT2, and WNT9B were positively correlated with six immune cells in LUSC, while WNT7B was negatively correlated. Our study indicated that WNT2B and WNT7A might have prognostic value in LUAD, and both of them might be important prognostic factors in LUSC and correlated to immune cell infiltration in LUAD and LUSC to a certain extent. Considering the prognostic value of WNT2B and WNT7A in NSCLC, we validated their mRNA and protein expression levels in NSCLC by performing qRT-PCR, western blot, and immunohistochemical staining on NSCLC pathological tissues and cell lines. This study may provide some direction for the subsequent exploration of the prognostic value of the WNTs and their role as biomarkers in NSCLC.

## Introduction

Lung cancer has a high incidence rate, accounting for 11.4% of cases and 18% of cancer mortality worldwide. By 2020, it has been the second most common cancer and the leading cause of death worldwide. There was little progress in its early screening and survival (1, 2). Non-small cell lung cancer (NSCLC) is the most common histological subtype in lung cancer patients, about 85% of these patients. It can be mainly divided into lung adenocarcinoma (LUAD), squamous cell lung cancer (LUSC), and large cell lung carcinoma, of which LUAD and LUSC are the most common (3, 4). For NSCLC therapeutic targets, molecular therapy, immune checkpoint inhibition, and lung cancer vaccination have made many advances (5–7). Nonetheless, lung cancer still has a 5-year survival rate lower than all other cancer types (8).

The Wingless-type (WNT) protein family consists of many cysteine-rich secreted glycoproteins, with a molecular weight of about 40 kDa, and 19 WNT genes have been identified in the human genome (9). WNT binds to members of the Frizzled (Fzd) receptor family on the cell surface to activate downstream effectors inside the cell, activating signal transduction cascades through three different pathways in the organism, i.e., the canonical WNT pathway, the non-canonical WNT/planar cell polarity (PCP) pathway, and the non-canonical WNT/calcium pathway (10). At the cellular level, WNT proteins participate in the regulation of cell proliferation, cell morphology, cell motility, and cell fate, playing an extensive role in organism development; moreover, dysregulation of WNT signaling also works during tumor formation (11, 12). WNT signaling serves as a molecular rheostat in organisms and participates in multiple physiological processes, including embryonic development, lineage commitment, adult stem cell homeostasis, and tissue regeneration (12). The transmission of WNT signaling and the generation and secretion of WNT ligands involve specialized and complex mechanisms (11–13). Conduction of WNT signaling has been demonstrated in many types of cancer (12), including but not limited to colorectal cancer (14), liver cancer (15), prostate cancer (16), skin cancer (17), melanoma (18) and lung cancer (12, 13). The WNT pathway plays an important biological role and represents prognostic value in NSCLC, and β-catenin produced by downstream activation of WNT ligands is indispensable in NSCLC development (12, 13, 19). Activation by WNT ligands in the canonical WNT pathway increases the concentrations of β-catenin in the cytoplasm, which binds to transcription factors of the T-cell factor (TCF)/Lymphoid enhancer-binding factor (LEF) family in the nucleus so as to drive transcription of WNT/β-catenin target genes. Some genomic data also suggest that an important component in lung cancer development is intrinsic WNT signaling within cancer cells (12). However, comprehensive studies on the expression and prognosis of the WNT family in NSCLC or its subtypes are lacking.

Our study used a publicly available tumor database to perform bioinformatics analysis of WNT gene family expression, prognosis, and immune infiltration in LUAD and LUSC so as to find reliable biomarkers and detailed prognosis of the WNT family in NSCLC.

## Materials and methods

### ONCOMINE database

ONCOMINE (https://www.ONCOMINE.org) is an online microarray database that allows differential expression analysis in many types of cancer (20). In this study, we compared the mRNA transcript expression differences of WNT genes by using Student’s t-test in this database, and the cut-off values of *p* value and fold-change were as follows: *p* value of 1E-4, fold-change of 2, gene rank of 10%.

### Tumor immune estimation resource (TIMER)

TIMER (https://cistrome.shinyapps.io/timer/) is an online tool that can systematically assess the infiltration level of immune cells in different cancer types and their clinical impact (21). In the “DiffExp” module, the expression level distribution of WNTs was displayed using boxplots, and the statistical significance of differential expression was assessed using the Wilcoxon test. Scatter plots representing the correlation of infiltration levels between WNT genes and the six immune cells were obtained by the “Gene” module. Then, by using the “Survival” module, the clinical correlation between WNTs and tumor immunity was explored by Cox regression analysis, including risk ratio and statistical significance. The level of tumor invasion between WNTs and tumors with different somatic copy number alterations was compared using the Wilcoxon rank-sum test in the “SCNA” module defined by GISTIC2.0. The heatmap of the correlation between WNTs and immune scores was created using the R software v4.0.3 package pheatmap (v1.0.12). The lollipop chart using the R software v4.0.3 package GSVA (v1.34.0) built-in algorithm ssGSEA to verify the relationship between WNTs expression and immune cells. The dataset used contained mRNA sequence data from 516 lung adenocarcinomas and 501 squamous cell lung cancers from TCGA tumors.

### UALCAN database

UALCAN (http://ualcan.path.uab.edu) is a comprehensive network resource that is taken from publicly available cancer genomics data (TCGA, MET500, and CPTAC) (22). In this study, the mRNA transcript expression and methylation data of WNT gene family were obtained by using the expression analysis module and methylation level module of UALCAN.

### Gene expression profiling interactive analysis (GEPIA)

GEPIA (http://gepia.cancer-pku.cn) is a web-based data mining platform with large RNA sequencing data from TCGA and GTEx (23). “Pathological Stage Plot” in the “ExpressionDIY” function is used for the correlation study of the pathological stage of WNTs in NSCLC patients. “Multiple Gene Comparison” module was used to obtain WNTs relative expression data, and matrix plots were created by the R v4.0.3 software package ggplot2 (v.3.3.5). Gene expression correlation analysis was performed in the “Correlation” module.

### Prognostic analysis

From the TCGA dataset (https://portal.gdc.com), to obtain raw counts and corresponding clinical information of RNA sequencing data of NSCLC tumors. For Kaplan-Meier (KM) curves, *p* values and hazard ratios (HR) with 95% confidence intervals (CI) were derived by log-rank test and univariate Cox proportional hazards regression, *p* < 0.05 was considered statistically significant. The above analysis method was created by the R software v4.0.3 package ggplot2 (v.3.3.5). The survival analysis results were verified by the Kaplan-Meier plotter (https://kmplot.com/) (24), the microarray database included caBIG, GEO, and TCGA. We obtained KM survival plots with hazard ratios and log-rank *p* values by univariate Cox regression analysis. For prognostic models of prognosis-related genes and clinical factors, univariate and multivariate Cox proportional hazards analyses were first performed, with risk scores including age, gender, pTNM stage of the tumor, and expression levels of prognosis-related genes. Based on the univariate and multivariate Cox regression analysis results, variables with significant differences in prognosis were extracted to construct a nomogram and predict the 1-year, 3-year, and 5-year survival rates of NSCLC patients. The above analysis was created using the R software v4.0.3 packages forestplot (v2.0.0) and rms (v6.2-0).

### cBioPortal database

The portal (https://www.cbioportal.org/) simplifies molecular profiling data from cancer tissues and cell lines into easily understood genetic, epigenetic, gene expression and proteomics events. It contains large cancer genomics datasets and has functions such as visualization, download and analysis (25). Based on the TCGA database, we analyzed 586 lung adenocarcinoma samples (TCGA, Firehose Legacy) and 511 squamous cell lung cancer samples (TCGA, Firehose Legacy) obtained from cBioPortal. mRNA expression z-scores (RNA Seq V2 RSEM) enter a z-score threshold ± 2.0, and protein expression z-scores (RPPA) enter a z-score threshold ± 2.0. Pearson’s correlation coefficient and *p* value were obtained by the “Co-expression” module, and the correlation heatmap between genes was constructed using the R software v4.0.3 package pheatmap (v1.0.12).

### Gene ontology (GO) and Kyoto encyclopedia of genes and genomes (KEGG) analysis

GO enrichment analysis examined the function of the WNT gene family from three levels: Biology Process, Molecular Function, and Cellular Component. KEGG is then used for pathway analysis. Histogram is implemented by R software v4.0.3 package clusterprofiler (v3.18.1) (26), the threshold is set as follows: *p* value cut-off of 0.05, q value cut-off of 0.05, minGSSize = 5, maxGSSize = 5000, The *p* value correction method uses Benjamini-Hochberg (BH). Protein-protein interaction (PPI) networks were constructed by STRING and Cytoscape. STRING database (https://string-db.org/) is designed to collect, score, and integrate all publicly available sources of protein-protein interaction information, supplemented by computational predictions (27). We constructed a protein interaction network of WNTs by the “Multipleproteins” function in STRING and performed GO and KEGG analyses.

### TRRUST

TRRUST (https://www.grnpedia.org/trrust/) is a TF-target interaction database based on text mining, including 8444 regulatory interactions of 800 transcription factors (TFs) in humans (28). A list of genes is entered on this website to query key TFs.

### TISIDB

TISIDB (http://cis.hku.hk/TISIDB/index.php) is a web portal containing multiple heterogeneous data on tumor and immune system interactions (29). We used TISIDB to reveal the relations between the abundance of tumor-infiltrating lymphocytes (TILs) and the expression of WNTs.

### Lung tissue samples

During January and March 2022, we collected 20 pairs of NSCLC tissues and adjacent normal tissues from the Third Xiangya Hospital of Central South University. These tissues were used to detect the expression levels of WNTs mRNA by quantitative real-time PCR (qRT-PCR). The Ethics Committee of the Third Xiangya Hospital of Central South University has approved the study. The approval number is I-22054.

### Cell culture

Human normal lung epithelial cell line (BEAS-2B), LUAD cell line (PC9) and LUSC cell line (NCI-H520) were obtained from American Type Culture Collection (ATCC) (Manassas, VA, USA). All cell lines were cultured in DMEM (Gibco, Carlsbad, CA) supplemented with 10% FBS (Gibco) and 1% pen/strep (Gibco) at 5% CO_2_ and 37°C.

### Quantitative real-time PCR (qRT-PCR)

According to the manufacturer’s instructions, total RNA was isolated from tissues or cells using Trizol reagent (TaKaRa, Japan). One microgram of RNA was reverse transcribed into cDNA using the Revert Aid First Strand cDNA Synthesis Kit (Thermo, USA). Quantitative RT-PCR was then performed with Pro Taq HS Premix Probe qPCR Kit (Accurate, Hunan, China). The amplification program consisted of one cycle of predenaturation at 95°C for 5 min, 37 cycles of denaturation at 95°C for 30 s, annealing at 61°C for 30 s, and extension at 72°C for 10 min. The GAPDH gene was used as an endogenous control gene for normalizing the expression of target genes. Each sample was analyzed in triplicate. Primer sequences are shown in **Supplementary Table S1**.

### Western blot

RIPA lysis buffer (Thermo Fisher Scientific, USA) containing protease and phosphatase inhibitors was used to lyse tissues. Protein lysates were separated by SDS-PAGE gels (Thermo Fisher Scientific, USA), blotted onto PVDF membrane (Roche, Switzerland) for analysis and incubated at 4°C overnight with the following primary antibodies: anti-WNT2B antibody (1:1000 dilution; Bioss, China), anti-WNT7A antibody (1:1000 dilution; Bioss, China), and anti-GAPDH antibody (1:2000 dilution; Cell Signaling Technology, USA). The results of the western blot analyses were performed with Image J software.

### Immunohistochemical staining

Immunohistochemistry was performed in 20 NSCLC tissues and adjacent normal tissues. Paraffin-embedded sections were stained to determine the expression level of proteins. Sections were incubated overnight at 4°C with anti-WNT2B antibody (1:100 dilution; Bioss, China), or anti-WNT7A antibody (1:100 dilution; Bioss, China). After washing with phosphate-buffered saline (PBS), the slides were incubated with a goat anti-rabbit IgG secondary antibody conjugated to fluorescein isothiocyanate (ZSDB-BIO, China) for 30 mins. They were washed with PBS and then incubated with an antifade reagent (Invitrogen, USA). Finally, staining was observed using an Olympus CX41 fluorescence microscope (Olympus, Japan). The results of the analyses were performed with Image J software.

## Results

### mRNA transcription and methylation levels of the WNT gene family in LUAD and LUSC

**Figure 1:**
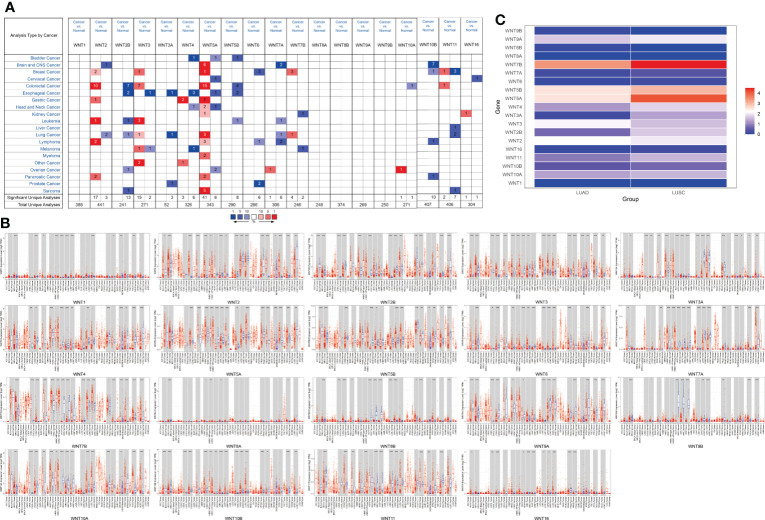
The mRNA transcription levels of the WNT gene family in LUAD and LUSC. **(A)** The mRNA transcription levels of WNT gene family in lung cancer (ONCOMINE). Red for overexpression, blue for downregulated expression. **(B)** Differential expression of WNT gene family in different tumor tissues and normal tissues (TIMER). Statistical significance of differential expression was assessed using the Wilcoxon test (*p* value significant codes: 0 ≤ *** < 0.001 ≤ ** < 0.01 ≤ * < 0.05 ≤. < 0.1). **(C)** The expression matrix plots for WNT gene family in LUAD and LUSC. Indicates the relative expression level among WNT genes.

Members of the WNT gene family and their respective specific chromosomal regions are presented in **Table 1**. We first examined the mRNA transcription of 19 WNT genes in lung cancer by the ONCOMINE database, as can be seen from **Figure 1A**. The detailed expression results in lung cancer are shown in **Table 2**, in which the expression levels of WNT2/2B/3A/7A/11 in lung cancer were significantly lower than those in normal tissues, while those of WNT3/5A/7B expressions were significantly higher, with altered expression levels of WNT5A and WNT7A in LUSC and the others are in LUAD.

**Table 1:** WNT gene family members and chromosome location.

HGNC ID (gene)	Approved symbol	Approved name	Previous symbols	Aliases	Chromosome
HGNC:12774	WNT1	WNT family member 1	INT1		12q13.12
HGNC:12780	WNT2	WNT family member 2	INT1L1	IRP	7q31.2
HGNC:12781	WNT2B	WNT family member 2B	WNT13	XWNT2	1p13.2
HGNC:12782	WNT3	WNT family member 3	INT4	MGC131950,MGC138321,MGC138323	17q21.31-q21.32
HGNC:15983	WNT3A	WNT family member 3A			1q42.13
HGNC:12783	WNT4	WNT family member 4		WNT-4	1p36.12
HGNC:12784	WNT5A	WNT family member 5A		hWNT5A	3p14.3
HGNC:16265	WNT5B	WNT family member 5B			12p13.33
HGNC:12785	WNT6	WNT family member 6			2q35
HGNC:12786	WNT7A	WNT family member 7A		WNT-7A	3p25.1
HGNC:12787	WNT7B	WNT family member 7B			22q13.31
HGNC:12788	WNT8A	WNT family member 8A		WNT8D	5q31.2
HGNC:12789	WNT8B	WNT family member 8B			10q24.31
HGNC:12778	WNT9A	WNT family member 9A	WNT14		1q42.13
HGNC:12779	WNT9B	WNT family member 9B	WNT15	WNT14B	17q21.32
HGNC:13829	WNT10A	WNT family member 10A			2q35
HGNC:12775	WNT10B	WNT family member 10B		WNT-12,SHFM6	12q13.12
HGNC:12776	WNT11	WNT family member 11			11q13.5
HGNC:16267	WNT16	WNT family member 16			7q31.31

**Table 2:** The significant changes of WNTs mRNA expression in transcription level between different types of Lung cancer.

Gene	Types of lung cancer *vs*. normal	Fold change	*p* value	t-Test	References
WNT2	Lung Adenocarcinoma *vs*. Normal	-2.089	3.63E-5	-4.473	Su Lung Statistics (30)
	Large Cell Lung Carcinoma *vs*. Normal	-3.134	1.53E-8	-8.323	Hou Lung Statistics (31)
WNT2B	Lung Adenocarcinoma *vs*. Normal	-2.063	1.77E-9	-8.935	Okayama Lung Statistics (32)
WNT3	Lung Adenocarcinoma *vs*. Normal	2.581	5.94E-10	9.100	Okayama Lung Statistics (32)
WNT3A	Lung Adenocarcinoma *vs*. Normal	-3.974	4.29E-30	-17.652	Selamat Lung Statistics (33)
WNT5A	Squamous Cell Lung Carcinoma *vs*. Normal	14.130	2.16E-7	6.311	Bhattacharjee Lung Statistics (34)
	Squamous Cell Lung Carcinoma *vs*. Normal	3.729	4.20E-6	6.292	Garber Lung Statistics (35)
	Squamous Cell Lung Carcinoma *vs*. Normal	3.897	4.95E-12	9.815	Hou Lung Statistics (31)
WNT7A	Small Cell Lung Carcinoma *vs*. Normal	-4.772	6.12E-5	-4.754	Bhattacharjee Lung Statistics (34)
WNT7B	Lung Adenocarcinoma *vs*. Normal	3.579	4.25E-14	10.960	Okayama Lung Statistics (32)
WNT11	Lung Adenocarcinoma *vs*. Normal	-2.512	4.12E-5	-4.540	Bhattacharjee Lung Statistics (34)
	Lung Adenocarcinoma *vs*. Normal	-3.138	1.31E-9	-7.752	Okayama Lung Statistics (32)

Additionally, we used TIMER to assess differences in expression levels of the WNT gene family in various cancers and normal tissues. As shown in **Figure 1B**, in LUAD, WNT3/5B/6/7B/8B/10A/10B/16 expressions significantly increased, WNT2/2B/3A/4/7A/9A/9B/11 expressions significantly decreased, and the results of WNT1 and WNT5A presented no statistical significance. In LUSC, the expressions of WNT2B/3/5A/5B/6/7B/10A/10B/16 were significantly promoted, while the expressions of WNT1/2/3A/7A/9A/9B/11 were significantly inhibited; the results of WNT4 and WNT8B were not statistically significant in LUSC, and WNT8A expression showed no statistical significance in LUAD and LUSC. **Figure 1C** showed the relative expression levels of WNT genes in LUAD and LUSC. Among the 19 WNT genes, WNT7B presented the highest relative expression level in LUAD, while the relative expression level of WNT5A/5B/7B was higher in LUSC than in LUAD, consistent with the above expression results.

**Figure 2:**
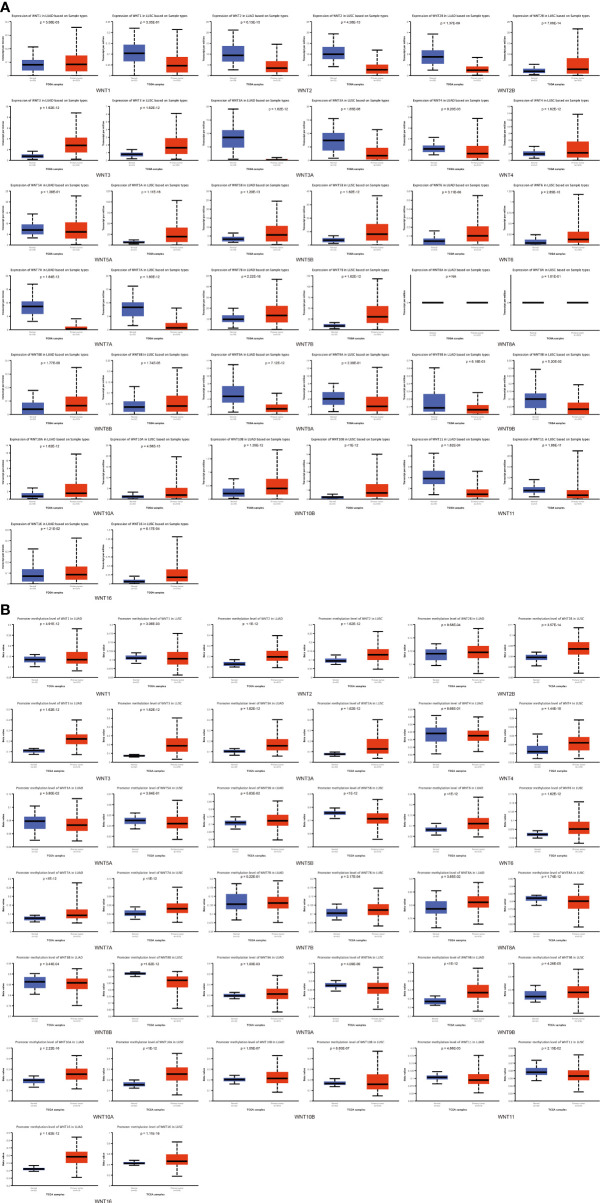
Compared with normal tissues, the WNT gene family’s mRNA expression and methylation levels in LUAD and LUSC (UALCAN). **(A)** The mRNA transcription levels of the WNT gene family in LUAD and LUSC. **(B)** The methylation level of the WNT gene family in LUAD and LUSC. *p* < 0.05 was considered statistically significant.

Moreover, UALCAN was used to explore the expression differences of the WNT gene family in normal tissues and in LUAD and LUSC, with the results shown in **Figure 2A**. In LUAD, the expression of WNT2/2B/3A/4/7A/9A/9B/11 was lower than that in normal tissues, while the expressions of WNT1/3/5B/6/7B/8B/10A/10B/16 were higher than that in normal tissues, presenting statistical significance; WNT5A and WNT8A results were not statistically significant; WNT2/3A/7A/11 were less expressed in LUSC than in normal tissues, while WNT2B/3/4/5A/5B/6/7B/8B/10A/10B/16 showed the opposite circumstance with statistical significance; WNT1/8A/9A/9B expressions were not statistically significant in LUSC. In summary, according to the comparison between databases, LUAD represented lessened WNT2/2B/3A/4/7A/9A/9B/11 expressions and enhanced WNT3/5B/6/7B/8B/10A/10B/16 expressions compared with normal tissues; in contrast, LUSC presented prohibited WNT2/3A/7A/11 expressions and promoted WNT2B/3/5A/5B/6/7B/10A/10B/16 expressions.

In addition, UALCAN was employed to explore the methylation levels of WNTs in LUAD and LUSC, with the results depicted in **Figure 2B**. In both LUAD and LUSC, the methylation levels of WNT1/2/2B/3/3A/6/7A/9B/10A/10B/16 were significantly improved, while those of WNT8B and WNT11 were significantly diminished. For WNT8A and WNT9A, their methylation levels were significantly boosted in LUAD but declined in LUSC. The methylation level of WNT5B significantly dropped in LUSC but not in LUAD, and that of WNT7B significantly went up in LUSC but not in LUAD.

### Prognostic value of WNTs in patients with LUAD and LUSC

**Figure 3:**
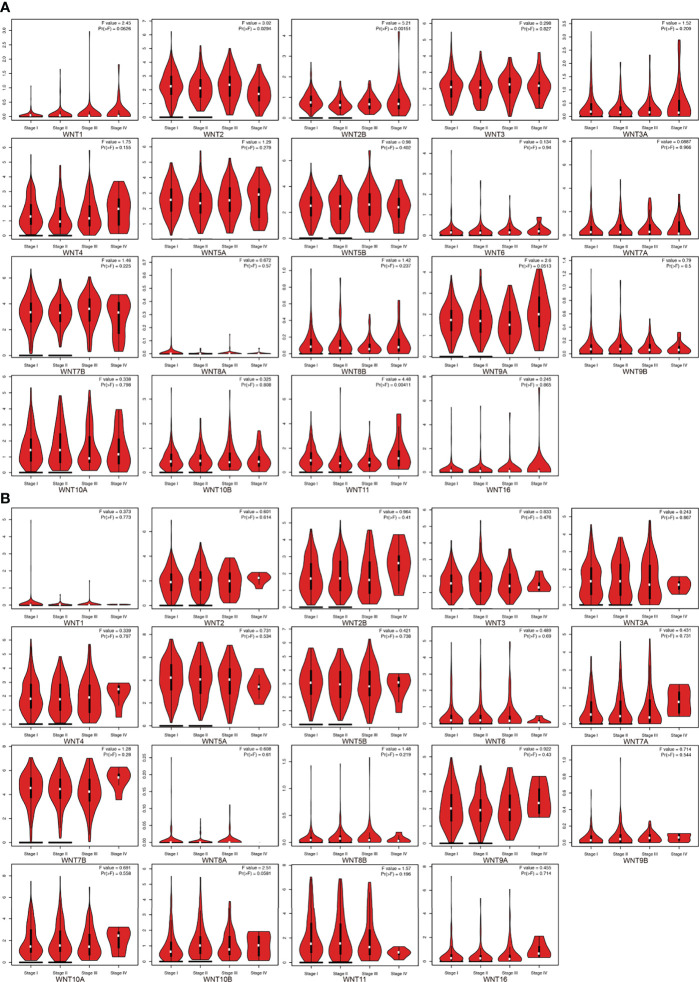
The mRNA expression of WNT gene family in different pathological stages of LUAD and LUSC (GEPIA). **(A)** Correlation between WNT gene family and pathological stage in patients with LUAD. **(B)** Correlation between WNT gene family and pathological stage in patients with LUSC.

This section first analyzed the correlation between the WNT gene and different pathological stages of LUAD and LUSC. As shown in **Figure 3A**, WNT2, WNT2B, and WNT11 were significantly correlated with pathological stages of LUAD patients, while WNTs presented no statistical significance in the correlation with LUSC (**Figure 3B**), indicating that the mRNA expression of WNT2, WNT2B, and WNT11 was significantly correlated with individual cancer stages of LUAD patients.

**Figure 4:**
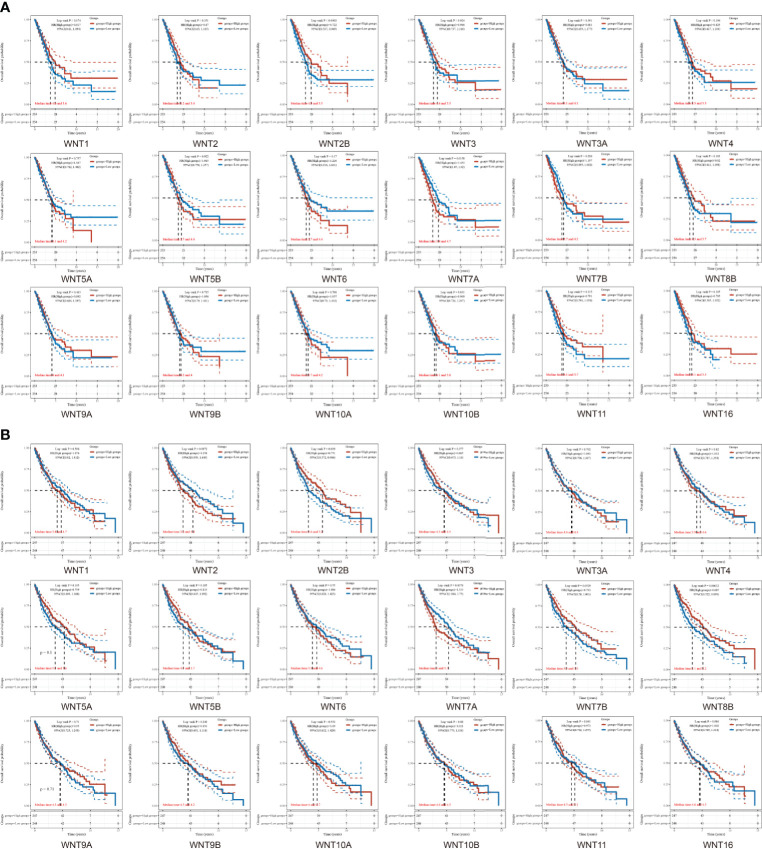
The overall survival (OS) curves of WNTs in LUAD and LUSC. **(A)** Kaplan-Meier survival analysis of WNTs in LUAD. **(B)** Kaplan-Meier survival analysis of WNTs in LUSC.

Next, data were extracted from The Cancer Genome Atlas (TCGA) dataset (https://portal.gdc.com). Raw counts and corresponding clinical information of RNA sequencing data were obtained from 516 LUAD patients and 501 LUSC patients for survival analysis. The clinical information of corresponding patients is shown in **Supplementary Table S2**. **Figures 4A, B** showed the overall survival (OS) curves in LUAD and LUSC. In LUAD patients, higher WNT2B expression and lower WNT7A expression were associated with a better prognosis. Similarly, higher expression of WNT2B and WNT8B and lower expression of WNT7A predicted better prognostic outcomes in LUSC patients. In addition, WNT8A expression was zero in more than half of the samples of LUAD and LUSC, which was thus not presented in this section. Then, the survival outcomes of WNTs in NSCLC were validated by the Kaplan-Meier plotter (**Supplementary Figure 1**), and higher WNT2B expression and lower WNT7A expression were associated with better prognostic outcomes in LUAD and LUSC, compatible with our results in TCGA. However, the results of WNT8B in LUSC were not significant, and thus WNT2B and WNT7A in LUAD and LUSC were selected for further analysis.

**Figure 5:**
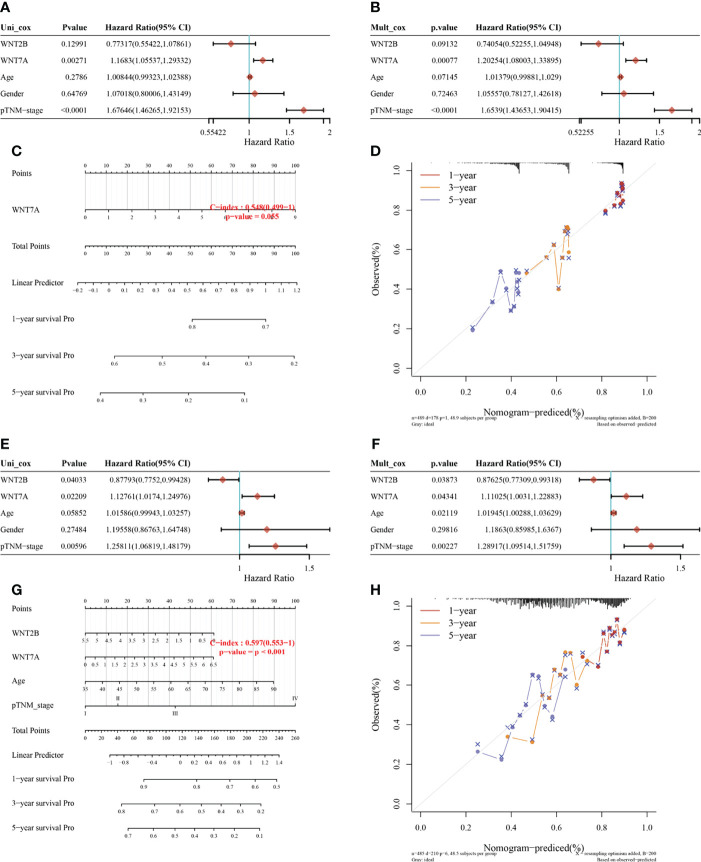
Univariate and multivariate Cox regression analysis of WNT gene expression and clinical characteristics and the nomogram predicting 1-y, 3-y, and 5-y survival in patients with LUAD and LUSC. **(A, B, E, F)** Hazard ratio and *p* value of constituents involved in univariate and multivariate Cox regression in LUAD **(A, B)** and LUSC **(E, F)**. **(C, G)** Nomogram to predict the 1-y, 3-y and 5-y overall survival of LUAD **(C)** and LUSC **(G)** patients. **(D, H)** Calibration curve for the overall survival nomogram model in the discovery group. The dashed diagonal line represents the ideal nomogram, and the red, orange and blue represent the 1-y, 3-y and 5-y observed nomograms. **(D)** for LUAD. **(H)** for LUSC.

For the prognosis-related genes obtained from the survival analysis, a prognostic model was constructed (**Figures 5A-H**) to explore their clinical impact on the disease process of patients. For the univariate (**Figure 5A**) and multivariate (**Figure 5B**) analysis of LUAD, WNT7A and pTNM stage were significantly different variables; in contrast, WNT7A was not statistically significant for the prediction of the LUAD process (**Figure 5C**). The univariate (**Figure 5E**) and multivariate (**Figure 5F**) analyses of LUSC indicated that WNT7A, WNT2B, and pTNM stage were significantly different variables. **Figure 5G** illustrated that WNT7A and WNT2B were statistically significant in predicting the LUSC process. Taken together, our results showed that WNT2B and WNT7A might have prognostic value in LUAD, and both of them might be independent prognostic factors for LUSC patients.

### Genetic alterations and association analysis of the WNT gene family in LUAD and LUSC patients

**Figure 6:**
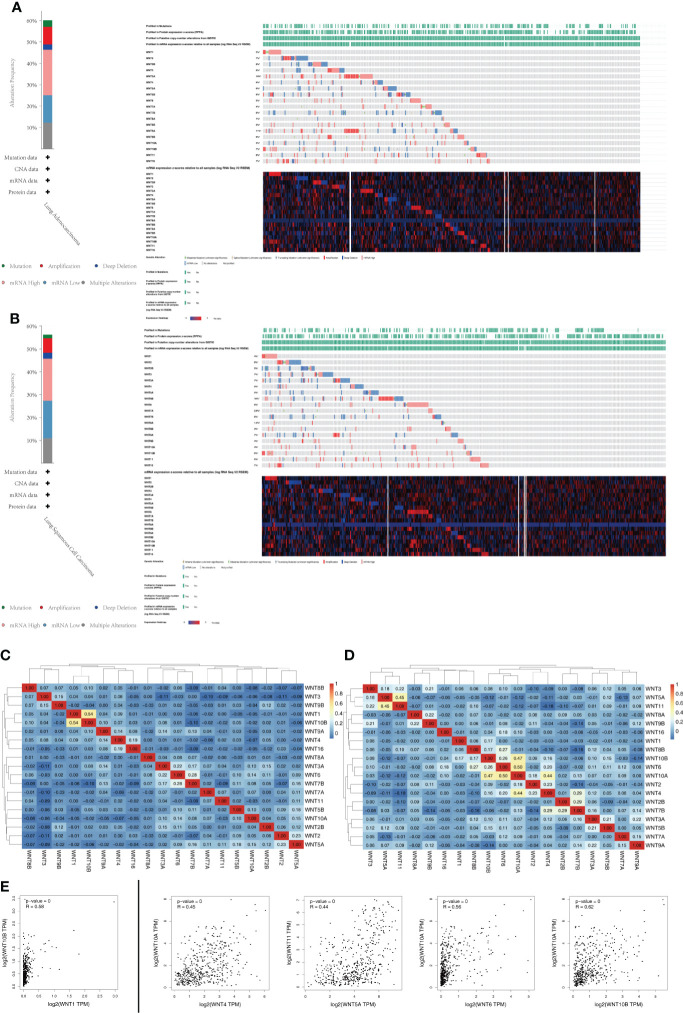
Genetic alterations and association analysis of the WNT gene family in NSCLC patients. **(A)** Genetic alterations of the WNT Gene Family in LUAD (cBioPortal). **(B)** Genetic alterations of the WNT Gene Family in LUSC (cBioPortal). **(C, D)** Correlation analysis heatmap between different WNT genes in LUAD **(C)** and LUSC **(D)**. **(E)** Correlation analysis between different WNT genes (GEPIA).

Through the cBioPortal online tool, we obtained the LUAD (TCGA, Firehose Legacy, http://gdac.broadinstitute.org/runs/stddata__2016_01_28/data/LUAD/20160128/) and the LUSC (TCGA, Firehose Legacy, http://gdac.broadinstitute.org/runs/stddata__2016_01_28/data/LUSC/20160128/) datasets from TCGA to analyze the genetic alterations and associations of WNT genes in NSCLC patients. As shown in **Figures 6A, B**, the WNT gene family represented different degrees of mutations in LUAD and LUSC. The mutation rate of WNT9A was the highest (11%) in LUAD (**Figure 6A**), while that of WNT5B was the highest (10%) in LUSC (**Figure 6B**). mRNA High and mRNA Low were the most common genetic changes in LUAD and LUSC.

In addition, Pearson’s correlation coefficients of relevant genes were obtained by the analysis of mRNA expression (RNA Seq V2 RSEM) samples (**Figures 6C, D**) and visualized by R software. A moderate correlation was observed between WNT1 and WNT10B (*p* value = 2.07e-40) in LUAD (**Figure 6C**), as well as between WNT4 and WNT10A (*p* value = 8.53e-25), WNT5A and WNT11 (*p* value = 5.96e-26), WNT6 and WNT10A (*p* value = 1.99e-32), and WNT10A and WNT10B (*p* value = 3.81e-29) in LUSC (**Figure 6D**). A total of 19 members of the WNT gene family were analyzed by the Correlation Analysis module of GEPIA, and similar correlation expression results were obtained, as shown in **Figure 6E**. In LUAD, WNT1 was moderately correlated with WNT10B (R = 0.58, *p* value = 0). However, In LUSC, moderate correlation was observed between WNT4 and WNT10A (R = 0.45, *p* value = 0), WNT5A and WNT11 (R = 0.44, *p* value = 0), and WNT6 and WNT10A (R = 0.56, *p* value = 0); moreover, a strong correlation was observed between WNT10A and WNT10B (R = 0.62, *p* value = 0).

### Functional enrichment and pathway analysis of the WNT gene family

**Figure 7:**
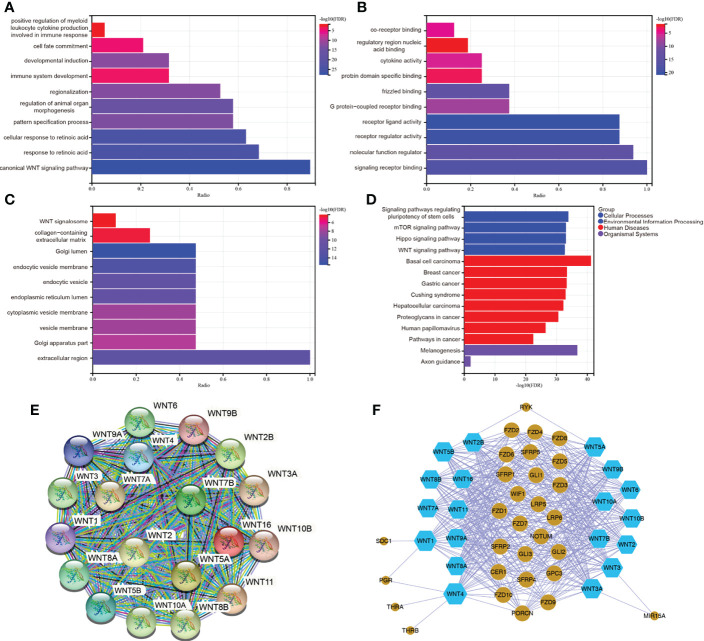
**(A–D)** GO and KEGG enrichment analysis of WNT gene family. **(E, F)** Protein-protein interaction network of WNT gene family. **(E)** for STRING. **(F)** for Cytoscape.

To further predict the functional role of the WNT gene family, we performed GO enrichment and KEGG pathway analysis of WNT gene family members with differential expression. **Figure 7A** showed that, in Biological Process, WNT genes were mainly enriched in canonical WNT signaling pathway, cellular response to retinoic acid, pattern specification process, regulation of animal organ morphogenesis, regionalization, immune system development, developmental induction, cell fate commitment, positive regulation of myeloid leukocyte cytokine production involved in immune response, etc. In the part of Molecular Function (**Figure 7B**), WNTs were mainly enriched in signaling receptor binding, molecular function regulator, receptor regulator activity, receptor ligand activity, G protein-coupled receptor binding, Fzd binding, protein domain specific binding, cytokine activity, regulatory region nucleic acid binding, co-receptor binding, etc. Additionally, WNT genes in Cellular Component (**Figure 7C**) were mainly enriched in extracellular region, Golgi apparatus part, vesicle membrane, cytoplasmic vesicle membrane, endoplasmic reticulum lumen, endocytic vesicle, endocytic vesicle membrane, Golgi lumen, collagen-containing extracellular matrix, WNT signalosome, etc. Subsequently, KEGG pathway enrichment analysis (**Figure 7D**) identified that WNT genes were mainly enriched in Signaling pathways regulating pluripotency of stem cells, mTOR signaling pathway, Hippo signaling pathway, WNT signaling pathway, Basal cell carcinoma, Breast cancer, Gastric cancer, Cushing syndrome, Hepatocellular carcinoma, melanogenesis, etc.

The PPI network analysis graph was constructed by STRING (**Figure 7E**) and Cytoscape (**Figure 7F**). STRING contained 19 nodes and 171 edges, PPI enrichment *p* value < 1.0e-16. As can be seen in **Figure 7F**, members of the WNT gene family had a close relationship with the Fzd receptor family, lipoprotein receptor-related protein (LRP) family, secreted Fzd-related proteins (SFRPs), and WNT inhibitory protein (WIF) family. Taken together, the functional enrichment and pathway analysis results for WNTs were as we know. The biological functions of the WNT gene family were mainly related to the canonical and non-canonical WNT signaling pathways, G-protein-coupled receptor binding, immune system development, and partial regulation of immune response.

### Transcription factor analysis of the WNT gene family in LUAD and LUSC patients

Compared with that of normal tissues, the transcriptional expression of WNTs was significantly altered in both LUAD and LUSC patients. Since transcription factors were involved in the human transcription initiation, we used the TRRUST database to explore the key transcription factors of WNT genes in humans. Among 19 WNT genes, WNT2, WNT2B, WNT3, WNT3A, WNT4, WNT5A, WNT7B, and WNT11 were included in the TRRUST database. We found that PITX2 was a key transcription factor associated with WNT gene regulation (**Table 3**).

**Table 3:** The key regulated factor of WNT genes in human.

Key TF	Description	List of overlapped genes	*p* value	Q value
PITX2	paired-like homeodomain 2	WNT2, WNT5A (36)	5.25E-05	5.25E-05

### Analysis of WNT gene family immune infiltration in LUAD and LUSC

**Figure 8:**
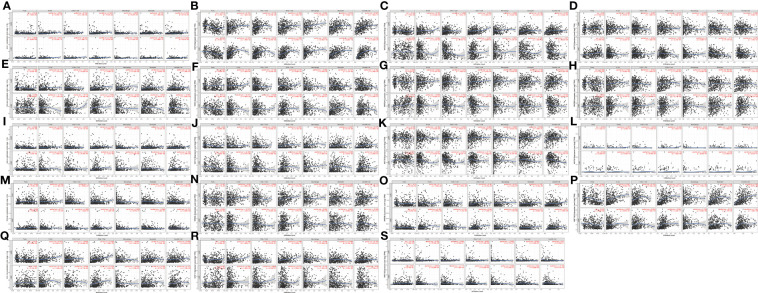
Correlation of WNT genes with immune cell infiltration in LUAD and LUSC (TIMER). **(A)** WNT1. **(B)** WNT2. **(C)** WNT2B. **(D)** WNT3. **(E)** WNT3A. **(F)** WNT4. **(G)** WNT5A. **(H)** WNT5B. **(I)** WNT6. **(J)** WNT7A. **(K)** WNT7B. **(L)** WNT8A. **(M)** WNT8B. **(N)** WNT9A. **(O)** WNT9B. **(P)** WNT10A. **(Q)** WNT10B. **(R)** WNT11. **(S)** WNT16.

**Figure 9:**
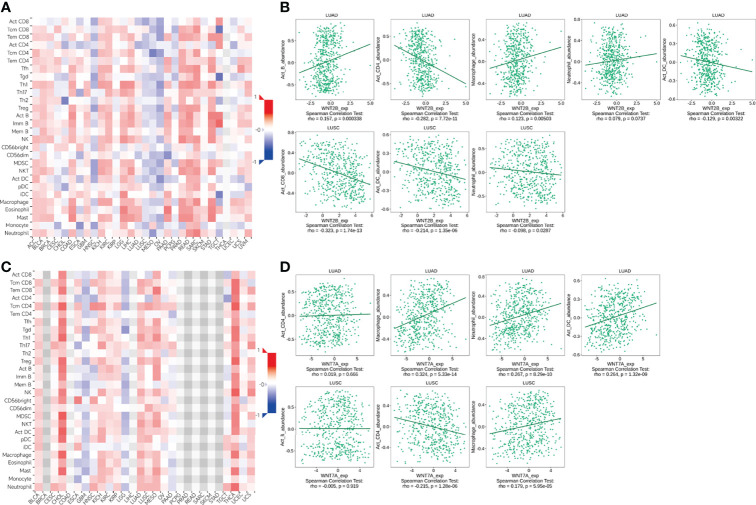
The association of WNT2B and WNT7A with immune cell infiltration (TISIDB). **(A, C)** The landscape of relationship between WNT2B **(A)** and WNT7A **(C)** expression and TILs in different types of cancer (red is positive correlated and blue is negative correlated). **(B)** Relationship between WNT2B and immune infiltration levels of B cell, CD4+ T cell, macrophage, neutrophil, dendritic cell in LUAD, and CD8+ T cell, neutrophil, dendritic cell in LUSC. **(D)** Relationship between WNT7A and immune infiltration levels of CD4+ T cells, macrophage, neutrophil, dendritic cell in LUAD, and B cell, CD4+ T cell, macrophage in LUSC.

Through the TIMER database, we explored the correlation between the WNT gene and immune cell infiltration (**Figure 8**). In LUAD, WNT2, WNT9B, and WNT10A represented a significantly positive correlation with six immune cells, including B cell, CD4+ T cell, CD8+ T cell, dendritic cell, macrophage, and neutrophil; WNT3 and WNT8B were positively correlated with tumor purity, while WNT1/2/2B/6/7A/7B/9A/9B/10A/10B/16 were negatively correlated with tumor purity. In LUSC, WNT1, WNT2, WNT7B, and WNT9B were significantly correlated with six immune cells, among which WNT1, WNT2, and WNT9B were positively correlated, and WNT7B was negatively correlated. However, the effect of WNTs on tumor purity in LUSC was slightly poorer than that in LUAD, and WNT3/5A/8B/11 were positively correlated with tumor purity, while WNT1/2/4/6/7A/98/10A were negatively correlated with tumor purity. For WNT2B and WNT7A, which might have prognostic value in LUAD and LUSC, as can be seen, in LUAD, WNT2B was positively correlated with all immune infiltration cells except CD8+ T cell; WNT7A was positively correlated with CD4+ T cell, macrophage, neutrophil and dendritic cell. In LUSC, WNT2B was negatively correlated with CD8+ T cell, neutrophil and dendritic cell; WNT7A was significantly correlated with B cell, CD4+ T cell and macrophage, among which CD4+ T cell and macrophage were positively correlated, and B cell was negatively correlated. Subsequently, we validated the association of WNT2B and WNT7A with immune cell infiltration by the TISIDB database. **Figures 9A, C** represented the relationship between WNT2B and WNT7A and tumor-infiltrating lymphocytes (TILs) in different cancer types. **Figure 9B** showed that, in LUAD and LUSC, except for CD4+ T cell and neutrophil, the correlation between WNT2B and other TILs infiltration levels was the same as the results obtained in TIMER. Similarly, **Figure 9D** represented that except for CD4+ T cell and B cell, the correlation between WNT7A and other TILs infiltration levels was the same as the results obtained in TIMER. Additionally, based on the dataset containing mRNA sequence data from TCGA, we constructed a heatmap of the correlation between WNTs and immune scores (**Supplementary Figure 2**), as well as a lollipop chart (**Supplementary Figure 3**) implemented by the ssGSEA algorithm to show the correlation between WNT2B and WNT7A and the level of immune cell infiltration, and these results are generally compatible with ours. Next, using a Cox proportional hazards model in TIMER, we explored the clinical relevance of WNTs with LUAD and LUSC tumor immunity, with the results shown in **Tables 4** and **5**. As can be seen, B cell (*p* < 0.001), WNT2B (*p* < 0.05), WNT3A (*p* < 0.05), WNT6 (*p* < 0.05) and WNT7A (*p* < 0.05) were significantly associated with clinical outcomes in LUAD (**Table 4**) but not in LUSC (**Table 5**).

**Table 4:** The Cox proportional hazard model of WNT genes with tumor-infiltrating immune cells in LUAD patients.

	Coef	HR	95%CI_l	95%CI_u	*p* value	Sig
B_cell	-5.084	0.006	0.000	0.099	<0.001	***
CD8_Tcell	0.564	1.758	0.297	10.414	0.534	
CD4_Tcell	2.396	10.976	0.599	201.099	0.106	
Macrophage	-0.007	0.993	0.066	15.018	0.996	
Neutrophil	-1.150	0.317	0.006	16.594	0.569	
Dendritic	-0.420	0.657	0.158	2.731	0.563	
WNT1	0.470	1.600	0.903	2.837	0.108	
WNT2	0.119	1.127	0.940	1.351	0.197	
WNT2B	-0.529	0.589	0.355	0.978	0.041	*
WNT3	-0.080	0.923	0.740	1.151	0.477	
WNT3A	-0.389	0.678	0.483	0.950	0.024	*
WNT4	-0.031	0.970	0.831	1.131	0.694	
WNT5A	-0.050	0.951	0.802	1.129	0.568	
WNT5B	-0.021	0.979	0.851	1.127	0.771	
WNT6	0.441	1.555	1.033	2.342	0.035	*
WNT7A	0.133	1.142	1.002	1.301	0.047	*
WNT7B	0.097	1.102	0.953	1.274	0.192	
WNT8A	-11.301	0.000	0.000	48.751	0.145	
WNT8B	-0.561	0.570	1.442	0.236	
WNT9A	0.060	1.062	0.847	1.332	0.601	
WNT9B	-0.251	0.778	0.252	2.407	0.663	
WNT10A	0.038	1.038	0.890	1.212	0.633	
WNT10B	0.140	1.150	0.762	1.735	0.506	
WNT11	0.030	1.030	0.859	1.236	0.749	
WNT16	-0.126	0.882	0.679	1.146	0.347	

*p* value significant codes: 0 ≤ *** < 0.001 ≤ ** < 0.01 ≤ * < 0.05 ≤. < 0.1.

**Table 5:** The Cox proportional hazard model of WNT genes with tumor-infiltrating immune cells in LUSC patients.

	Coef	HR	95%CI_l	95%CI_u	*p* value	Sig
B_cell	1.297	3.657	0.305	43.804	0.306	
CD8_Tcell	-1.087	0.337	0.055	2.086	0.242	
CD4_Tcell	0.575	1.777	0.124	25.500	0.672	
Macrophage	0.321	1.378	0.120	15.796	0.796	
Neutrophil	1.162	3.196	0.117	87.541	0.492	
Dendritic	0.244	1.277	0.304	5.360	0.739	
WNT1	0.336	1.399	0.956	2.047	0.084	·
WNT2	-0.003	0.997	0.851	1.166	0.966	
WNT2B	-0.130	0.878	0.764	1.010	0.068	·
WNT3	0.057	1.059	0.848	1.323	0.614	
WNT3A	0.012	1.012	0.877	1.168	0.871	
WNT4	-0.073	0.930	0.800	1.081	0.344	
WNT5A	0.009	1.009	0.896	1.136	0.878	
WNT5B	-0.103	0.902	0.801	1.016	0.088	·
WNT6	-0.049	0.952	0.776	1.169	0.639	
WNT7A	0.103	1.108	0.977	1.257	0.111	
WNT7B	-0.029	0.971	0.842	1.119	0.685	
WNT8A	3.795	44.478	0.026	76678.348	0.318	
WNT8B	-0.229	0.796	0.309	2.051	0.636	
WNT9A	0.022	1.022	0.879	1.189	0.772	
WNT9B	-1.721	0.179	0.032	0.997	0.050	·
WNT10A	0.092	1.096	0.969	1.240	0.146	
WNT10B	-0.015	0.985	0.834	1.163	0.855	
WNT11	0.018	1.018	0.920	1.127	0.728	
WNT16	0.010	1.010	0.848	1.202	0.913	

*p* value significant codes: 0 ≤ *** < 0.001 ≤ ** < 0.01 ≤ * < 0.05 ≤. < 0.1.

**Figure 10:**
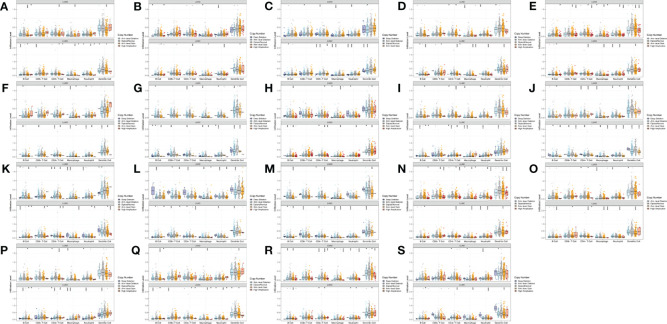
Comparison of tumor infiltration levels among tumors with different SCNA for WNTs in LUAD and LUSC samples (*p* value significant codes: 0 ≤ *** < 0.001 ≤ ** < 0.01 ≤ * < 0.05 ≤. < 0.1). **(A)** WNT1. **(B)** WNT2. **(C)** WNT2B. **(D)** WNT3. **(E)** WNT3A. **(F)** WNT4. **(G)** WNT5A. **(H)** WNT5B. **(I)** WNT6. **(J)** WNT7A. **(K)** WNT7B. **(L)** WNT8A. **(M)** WNT8B. **(N)** WNT9A. **(O)** WNT9B. **(P)** WNT10A. **(Q)** WNT10B. **(R)** WNT11. **(S)** WNT16.

Moreover, for the 19 genes of WNTs, we compared the tumor infiltration level with different degrees of somatic copy number alterations (SCNA) in LUAD and LUSC patients, with the results illustrated in **Figure 10**. We found that all significant copy number change types appeared in arm-level deletion, arm-level gain, deep deletion, and high amplification. In LUAD, WNT7B and WNT8A were significantly correlated with deep deletion and arm-level deletion of six immune cells, respectively, and WNT3A and WNT9A were significantly correlated with high amplification of six immune cells. In LUSC, WNT4, WNT5A, WNT7A, and WNT8B were significantly correlated with arm-level deletion of six immune cells; WNT3 and WNT9B were significantly correlated with the arm-level gain of six immune cells; WNT7B was significantly correlated with high amplification of six immune cells. For WNT2B and WNT7A, WNT2B is significantly correlated with arm-level deletion of CD8+ T Cell, CD4+ T cell, macrophage, neutrophil and dendritic cell in LUAD; in LUSC, WNT2B showed a significant correlation with CNA levels in all immune cells except for CD8+ T cells. Similarly, WNT7A showed a significant correlation with CNA levels of all immune cells in LUAD except for CD8+ T Cell; and WNT7A showed a significant correlation with CNA levels of all immune cells in LUSC.

### Validation of the mRNA and protein expression levels of WNT2B and WNT7A in LUAD and LUSC

**Figure 11:**
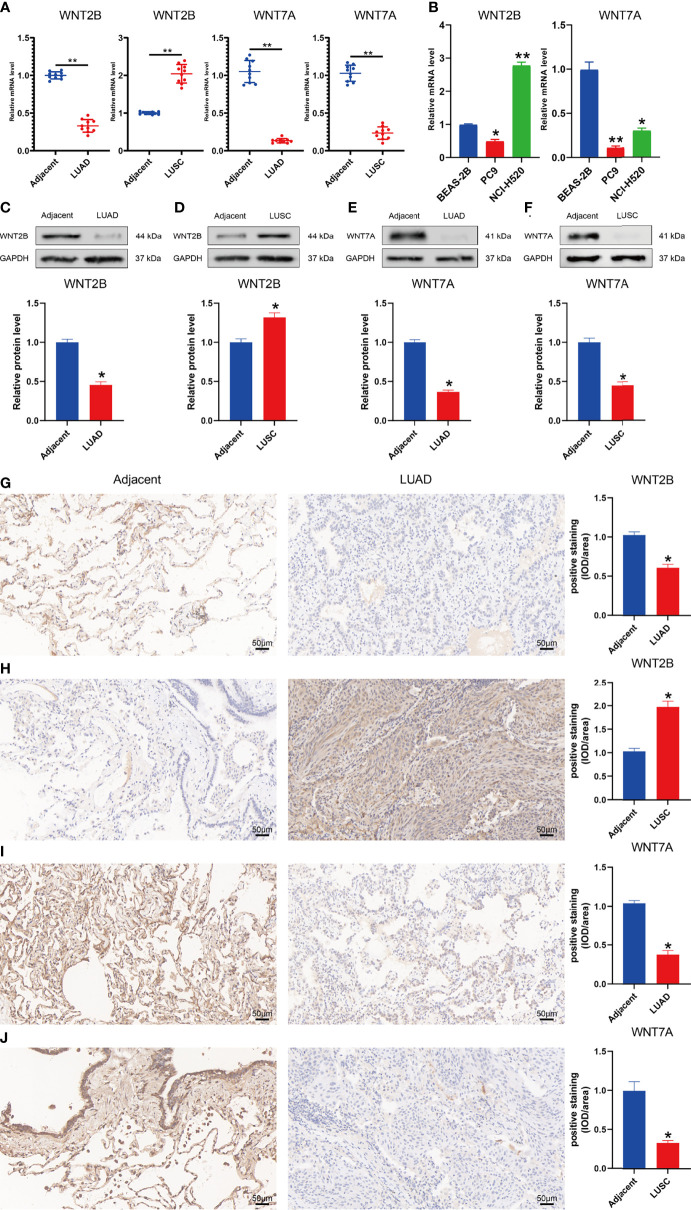
Validation of mRNA and protein expression levels of WNT2B and WNT7A in NSCLC. **(A)** Comparison of mRNA expression levels of WNTs in 20 paired NSCLC tissues and adjacent normal tissue samples by qRT-PCR. **(B)** Comparison of mRNA expression levels of WNTs in LUAD cell line (PC9) and LUSC cell line (NCI-H520) with human normal lung epithelial cell line (BEAS-2B). **(C-F)** The western blot analyses. **(G–J)** The immunohistochemical staining in 20 NSCLC tissues and adjacent normal tissues.

To validate the mRNA expression levels of WNT2B and WNT7A in adjacent normal tissues, LUAD tissues, and LUSC tissues, qRT-PCR was performed (**Figure 11**). The results indicated that the expressions of WNT2B and WNT7A were downregulated in tissue samples from 10 LUAD cases. Moreover, WNT2B expression was upregulated, while WNT7A expression was downregulated in tissue samples from 10 LUSC cases (**Figure 11A**). In addition, compared with normal lung epithelial cell line (BEAS-2B), WNT2B and WNT7A were significantly underexpressed in the LUAD cell line (PC9), WNT2B was significantly highly expressed in the LUSC cell line (NCI-H520), and WNT7A was significantly underexpressed in LUSC cell line (NCI-H520) (**Figure 11B**). These results were consistent with the results of bioinformatics analysis. Next, the protein expression levels of WNT2B and WNT7A were validated by western blot (**Figures 11C–F**) and immunohistochemical staining (**Figures 11G–J**), which were consistent with the mRNA expression levels.

## Discussion

At the beginning of this study, we obtain the complete mRNA expression of WNT in LUAD and LUSC through three databases. In LUAD patients, WNT2/2B/3A/4/7A/9A/9B/11 expressions decrease, and WNT3/5B/6/7B/8B/10A/10B/16 expressions increase. In contrast, in LUSC patients, WNT2/3A/7A/11 expressions are decreased, and WNT2B/3/5A/5B/6/7B/10A/10B/16 expressions are increased. As in our findings, WNT2 is overexpressed in NSCLC (37). A higher expression of WNT2 was associated with a worse survival prognosis in some studies (38). In addition, anti-WNT2 antibodies could induce specific apoptosis in NSCLC by inhibiting WNT signaling (37), making anti-WNT2 monoclonal antibodies one of the strategies for treating lung cancer. Robert A Winn et al. carried out cell experiments and found that the mRNA expression level of WNT7A was significantly reduced in NSCLC cells and primary tumors (39), consistent with our down-regulation found.

In addition, according to the methylation analysis of the UALCAN database, in LUAD, the methylation levels of WNT1/2/2B/3/3A/6/7A/8A/9A/9B/10A/10B/16 are significantly enhanced, while those of WNT8B and WNT11 are significantly reduced; in LUSC, the methylation levels of WNT1/2/2B/3/3A/4/6/7A/7B/9B/10A/10B/16 are significantly promoted, while those of WNT5B/8A/8B/9A/11 are significantly inhibited. Methylation of WNTs has been demonstrated to show a partial correlation with tumor development (40). Tae-Hyung Kim et al. investigated a correlation between WNT7A promoter methylation and loss of E-cadherin expression by detecting WNT7A promoter methylation in NSCLC patients (40). E-cadherin loss was closely related to the poor prognosis and survival of many cancers (41), suggesting that promoter methylation-induced WNT7A loss might be an important prognostic factor in NSCLC. The 3p25 is the chromosome location of the WNT7A (**Table 1**). Cytogenetic analysis reveals that 3p25 is a common genetic deletion site in lung cancer, and similar regions also include 3p21.3 and 3p14-cen (42). The elevated methylation levels of WNT7A may be associated with its loss of expression (43).

In the next prognostic value and survival analysis, we first explore the WNT expression at different pathological stages of LUAD and LUSC patients. With the LUAD development, the WNT2 expression decreases as a whole, reaching the lowest level when the tumor progresses to stage IV; the expression of WNT2B presents no obvious change but tends to decrease with the disease progression, of which a few patients show a sudden rise in the expression in stage IV; WNT11 showed an increase in the expression with the tumor development. The results indicate that WNT2/2B/11 may be able to predict the tumor progression of LUAD patients. Next, we obtain the prognostic outcomes shown by the WNT gene with different degrees of expression in NSCLC patients by KM survival curves, and the survival results are also validated by another open database. In our study, highly expressed WNT2B and lowly expressed WNT7A predict better prognosis in LUAD and LUSC patients. After regression analysis and screening, the prediction results of WNT7A during the LUAD are not significant enough, according to the significant differential results obtained by univariate and multivariate Cox analyses of RNA-seq data from LUAD samples in TCGA. Despite this, we believe that WNT2B and WNT7A may act the important prognostic values in LUAD and LUSC, based on the significant differences shown in KM curves. The nomogram provides some guidance for WNT7A and WNT2B in predicting disease progression and survival in LUSC patients clinically.

WNT7A bound to Fzd9 and promoted epithelial differentiation of NSCLC cells by activating the cJun N-terminal kinases (JNKs) pathway (44). Moreover, the low expression of WNT7A in NSCLC might play a role in lung cancer progression through its effect on E-cadherin transcription (45). WNT7A could also induce hypertrophy of muscle fibers by driving activation of the AKT/mTOR signaling pathway (46). In addition, WNT7A acted as an upstream signaling molecule of the WNT canonical pathway (47). Its overexpression could transcriptionally activate WNT target genes through the WNT/β-catenin signaling pathway, thus leading to cancer development, or it could bind to some WNT receptors to promote the malignant phenotype of cancer (48), partially corroborating our findings. Some studies have suggested that the up-regulated expression of WNT2B promoted the malignant behavior of NSCLC (49, 50). After methodological comparison, we believe that the reason for the difference in the above results with our study may be relevant to the different cell types of NSCLC. That is, the previous studies focus on all NSCLC patients; in contrast, in this study, the prognostic analysis for WNT2B is based on the clinical samples of LUAD and LUSC patients. In addition, in our results, the transcriptional expression of WNT2B is divergent in LUAD and LUSC patients, implying the different roles of WNT2B in tumor progression in LUAD and LUSC. More research is required to explore the lung cancer field where WNT2B is located, which may be a breakthrough point in subsequent studies. WNT8B is one of the typical WNT ligands, transcriptionally regulated by zinc finger transcription factor 191 (ZNF 191) and playing an important role in hepatocellular carcinoma (HCC) proliferation through the canonical WNT pathway (serving as a new prognostic marker for HCC) (51). Few related studies were found on WNT8B in lung cancer, which may provide some directions for future research. It is worth mentioning that in the existing studies, WNT7A and WNT2B played different roles in the prognosis of different cancer types, which may be attributed to their various mechanisms of action in diverse cancer cell lines and the activated signaling pathways. More critically, our study is the first to use clinical samples to link the WNT expression with different cell types in LUAD and LUSC, providing more insights and evidence for the subsequent research of the WNT gene family in the prognosis of LUAD and LUSC patients. We look forward to more subsequent extensive studies of WNTs in different cell types of NSCLC.

Immediately afterward, we explore the WNT gene mutation in LUAD and LUSC by cBioPortal. All WNT genes are detected to show different degrees of mutation, of which mRNA High and mRNA Low are the most common genetic changes in LUAD and LUSC. The mutations presented by WNTs showed significant phenotypes in mice, *Caenorhabditis elegans*, and *Drosophila* (10). Heparin-sulfated forms of proteoglycans (HSPG) were involved in the WNT transport and played a role in stabilizing WNT proteins or assisting their transmission between cells. In *Drosophila*, loss of HSPG or mutations in genes encoding enzymes that modify them could lead to the generation of phenotypes similar to wingless mutants (9). Since it is associated with the biological role of stem cells (9), WNT pathway mutations are very common in cancer, especially in tissues relying on WNT for self-repair (11). However, mutations in WNT pathway components frequently appeared in colorectal cancer (19, 52). Although some studies have shown that mutations in β-catenin and its pathway components caused downstream activation of WNT signaling in LUAD (53), this mutation was still rare in lung cancer (13, 54). Analysis of RNA-seq and miRNA-seq data from several NSCLC patients suggested that alterations in WNT9A might impact cancer progression by affecting the interference between lncRNAs, miRNAs, and mRNAs (55). WNT5B signaled through the non-canonical WNT signaling pathway, which was usually used as an antagonist of the canonical WNT signaling pathway and played a role in the proliferation, differentiation, and migration of cells (56). Pairwise WNT genes presenting tight links in correlation analysis might play a synergistic or similar role in cancer progression. And both WNT5A and WNT11 act through the non-canonical WNT/calcium signaling pathway. It was found that when the mRNA of WNT5A or WNT11 was injected into one-cell zebrafish embryos, both of the mRNA of WNT5A or WNT11 could double the frequency of calcium transients in the enveloping layer of the blastodisc (57). Similar to WNT11 (58, 59), WNT5A has also been shown to help to inhibit cell migration and invasiveness in cancer cell lines (60).

According to the functional enrichment and pathway analysis results, we can see that WNT genes are highly enriched in WNT signaling pathways, immune system development and Fzd receptor/G protein-coupled receptor binding, etc. These functions are highly correlated with the action mode of the WNT gene family in the body. For example, overexpression of WNT4, WNT5A, and WNT11 has been found to play a role in early embryo development through the non-canonical WNT signaling pathway (57, 61). As an important regulator of cell growth and differentiation, WNT5A has also been demonstrated to participate in the tumor growth inhibition through the non-canonical WNT/calcium signaling pathway (62). WNT11 acted as a non-canonical WNT pathway ligand and similarly activated the WNT/calcium signaling pathway (63). WNT signaling might also serve as an essential regulator of stem cell proliferation or self-renewal selection in the body. For example, WNT3A promoted the self-renewal of hematopoietic stem cells *in vitro* (9). It turned out that there was overexpression of some WNT pathway components and active WNT signaling in most NSCLC (13). Mark Shapiro et al. performed immunoblot analysis on tumor and normal lung tissues from several patients with stage I NSCLC after resection, confirming that activation of the WNT pathway was significantly associated with high tumor recurrence rates (64).

It is well-known that transcription factors play an important role in the transcription process in humans. Therefore, we query key WNT-associated transcription factors in humans. Paired-like homeodomain transcription factor 2 (PITX2) is one of the bicoid/paired-like homeobox gene family, which is located at position 4q25 of the chromosome and is a transcription factor involved in the anterior structure development (65). PITX2 could promote cancer progression by activating the WNT gene and regulating the WNT/β-catenin pathway (36). In addition, PITX2 was also found to be a target gene of LEF1 (66). During WNT/β-catenin pathway regulation, PITX2 could activate specific growth regulatory genes, such as cyclin D1, cyclin D2 and c-Myc Kioussi (67). Jing Luo et al. investigated the involvement of PITX2 in the WNT/β-catenin pathway in LUAD, showing that PITX2 was a potential up-regulated oncogene in LUAD, and PITX2 was overexpressed in LUAD and relevant to poor prognosis. They also found that PITX2 could activate the WNT/β-catenin pathway by enhancing WNT3A transcription, thereby exerting an oncogenic effect (68).

After this, we explore the association between the WNT gene family and six immune cells, including B cell, CD4+ T Cell, CD8+ T Cell, dendritic cell, macrophage, and neutrophil. The prognosis-related genes WNT2B and WNT7A are significantly correlated with immune cell infiltration and the infiltration level of different SCNAs in both LUAD and LUSC. WNT signaling has been demonstrated to regulate immune cell function, significantly inhibit the maturation and differentiation of T cells and dendritic cells (69), and alter the differentiation of CD4+ cells and CD8+ effector T cells (70, 71). WNT signaling plays an important role in the tumor immune microenvironment, and WNT signaling can promote naive T cell maturation and control the proliferation and survival of progenitor B-cells and B-1 cells (72, 73). Moreover, WNT signaling can also increase the level of β-catenin in malignant cells, which leads to the subsistence of Regulatory T cells (Tregs), differentiation of CD4 + T cells into Th17 subtypes and secretion of IL10 and IL12 in dendritic cells (74). In addition, the canonical WNT signaling pathway was identified as one of the important immune evasion-related oncogenic pathway signals (75). Therefore, relevant WNT signaling pathways have been increasingly recognized as a potential target for cancer therapy. NFAT is a transcription factor regulated by the calcium/calmodulin-dependent protein phosphatase calcineurin and a downstream factor of WNT5A, localizing in the cytoplasm of resting T cells (76, 77). NFAT could enhance the expression of IL-2, an important mediator of graft-versus-host disease, under the stimulation of T cells, while cyclosporine A (CsA) acted as an immunosuppressive agent by reducing the nuclear accumulation of NFAT and then IL-2 expression (78). However, in the presence of CsA, T cell treatment with WNT5A resulted in nuclear accumulation of NFAT, enabling WNT5A to lead to CsA resistance through the WNT/heterotrimeric GTP-binding protein pathway (57). Through the B cell proliferation in WNT5A-deficient mice, WNT5A was found to inhibit B cell proliferation through WNT/calcium to act as a tumor suppressor (62). Immune escape has been a key in cancer research in recent decades, and the advent of treatments such as molecular therapy and immune checkpoint inhibition for cancer has also shed light on the conquest of lung cancer (5–7). Overall, based on the results in TIMER and TISIDB, our results suggest that there may be a degree of correlation between WNT2B and WNT7A and the level of immune cell infiltration. Based on limitations such as study objectives and experimental samples, etc, we did not conduct further experiments to verify the relationship between WNTs and immune cells, therefore, this field still needs to be explored through a large number of experiments. Furthermore, because of new drug resistance mechanisms, gene polymorphisms, and other reasons, many studies are still expected to support the research and treatment methods in this field.

Finally, we validate the mRNA and protein expression levels of WNT2B and WNT7A in NSCLC by qRT-PCR, western blot, and immunohistochemical staining, obtaining consistent experimental results with our analysis, further increasing the credibility and accuracy of our conclusions.

In summary, our study provides some insights into the WNT gene family in NSCLC patients about differential expression profile, potential prognostic value, immune infiltration level, etc. Nevertheless, this study still has some limitations. First, we did not perform the gene knockdown or overexpression of WNT2B and WNT7A to investigate the effects of WNT2B and WNT7A on prognostically relevant functions such as proliferation and migration of NSCLC, which will be the research focus of our team in the next phase. Second, based on existing studies, the role of some WNT genes in cancer conduction is not exhaustive, and the detailed mechanism of these genes still needs numerous subsequent studies. Our research is expected to provide further exploration direction and reference significance for subsequent research in this field.

## Conclusion

This paper, for the first time, explored the comprehensive situation of the WNT gene family in LUAD and LUSC by bioinformatics methods and the differences of the WNT gene family in LUAD and LUSC fields. According to the survival analysis results, WNT2B and WNT7A might have prognostic value in LUAD, and both of them might be important prognostic factors for LUSC patients. In addition, WNT2B and WNT7A might show some correlation with immune cell infiltration of LUAD and LUSC, but their specific prognosis and immune infiltration-related mechanisms need further experimental validation. This study may provide some direction for the subsequent exploration of the biomarker or prognostic value of the WNT gene family in NSCLC.

## Data availability statement

The original contributions presented in the study are included in the article/**Supplementary Material**. Further inquiries can be directed to the corresponding author.

## Ethics statement

The studies involving human participants were reviewed and approved by the ethics committee of the Third Xiangya Hospital of Central South University. Written informed consent for participation was not required for this study in accordance with the national legislation and the institutional requirements.

## Author contributions

WL and MT designed the study. JW and QY prepared the manuscript. All authors contributed to the article and approved the submitted version.

## Funding

This study was supported by the Hunan Province Natural Science Foundation (No. 2021JJ40939, No. 2022JJ40253), the Scientific research project of Hunan Health Commission (No. 202203014949, No. 202102041763, No. 20200985), the Changsha Municipal Natural Science Foundation (No. kq2014267), and the Hunan Cancer Hospital Climb Plan (No. 2020QH001).

## Conflict of interest

The authors declare that the research was conducted in the absence of any commercial or financial relationships that could be construed as a potential conflict of interest.

## Publisher’s note

All claims expressed in this article are solely those of the authors and do not necessarily represent those of their affiliated organizations, or those of the publisher, the editors and the reviewers. Any product that may be evaluated in this article, or claim that may be made by its manufacturer, is not guaranteed or endorsed by the publisher.
