# Supplementary figures and images for: Validation and analysis of expression, prognosis and immune infiltration of WNT gene family in non-small cell lung cancer
Source: Front Oncol. 2022 Jul 25;12:911316. doi: 10.3389/fonc.2022.911316 (PMC9359207; doi:10.3389/fonc.2022.911316)

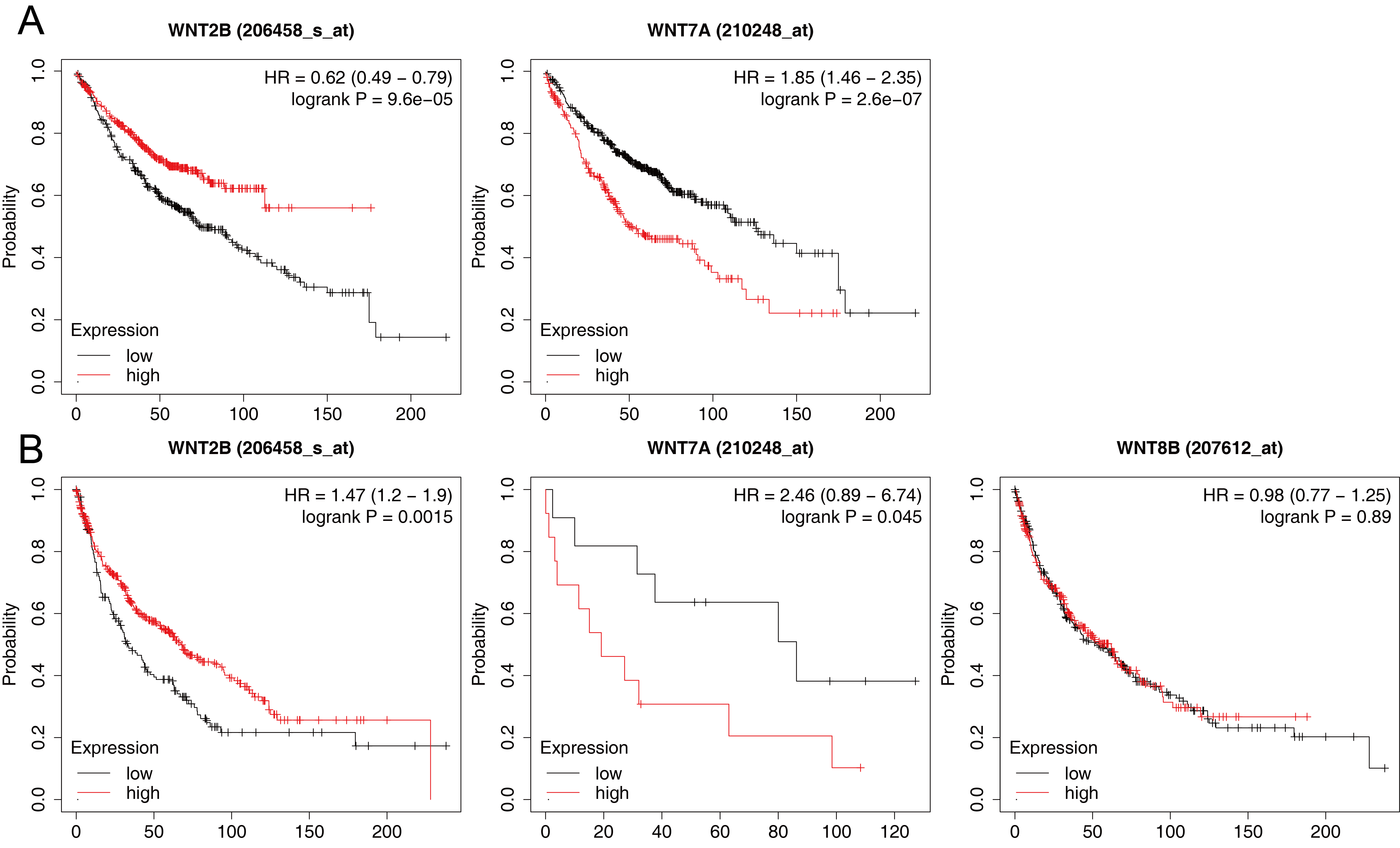

Supplement: Supplementary file 1 [file Image_1.jpeg]

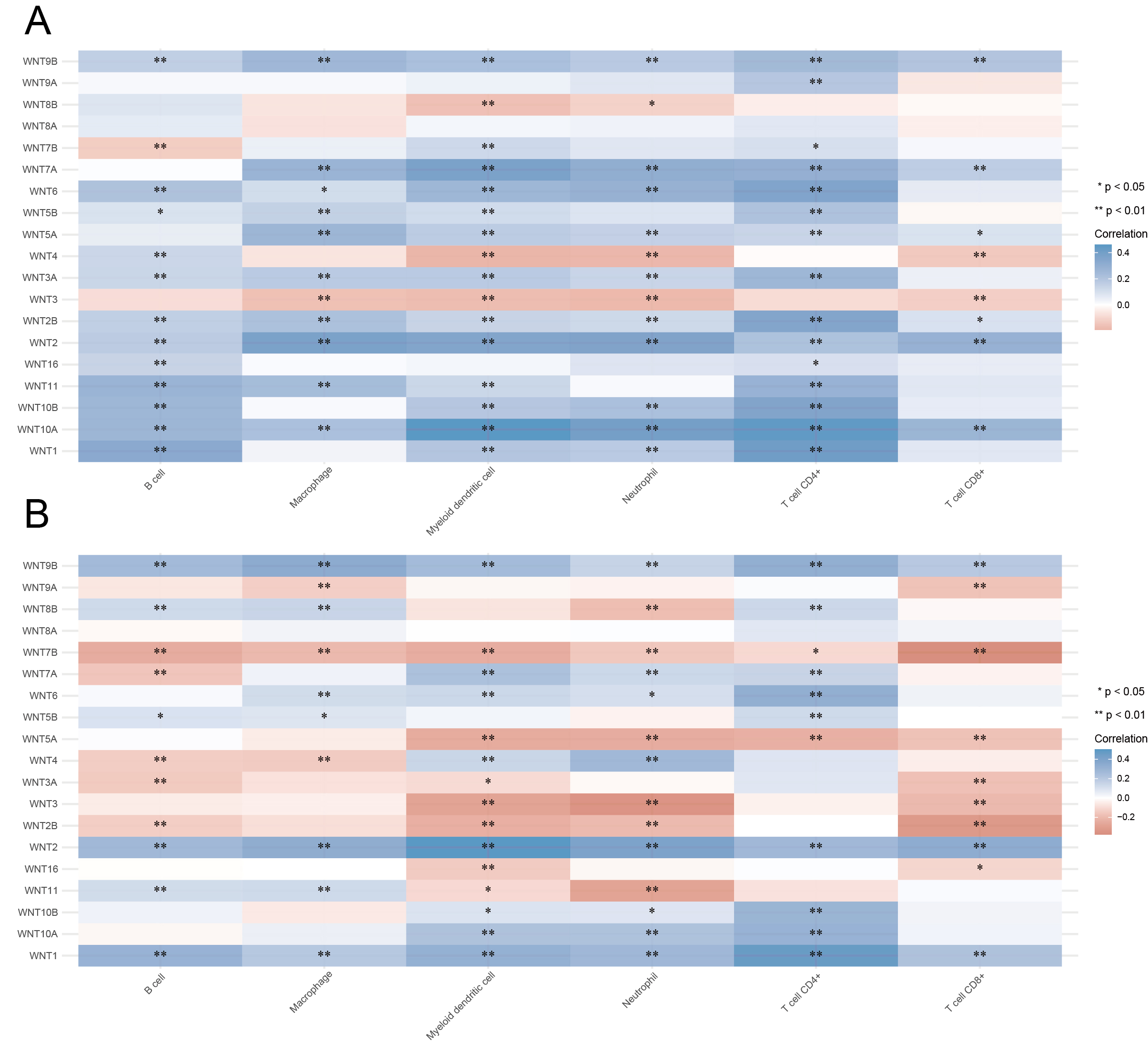

Supplement: Supplementary file 2 [file Image_2.jpeg]

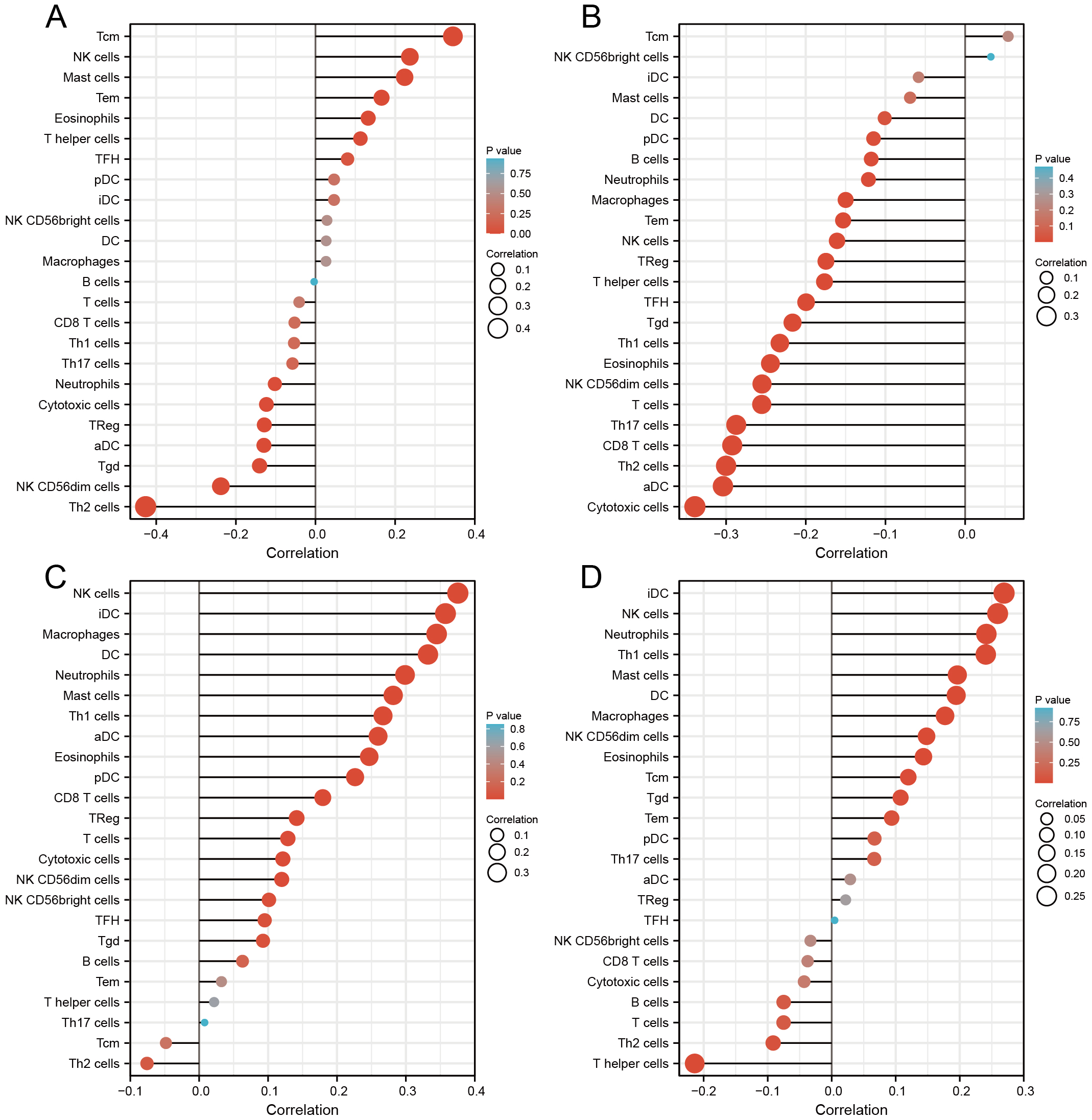

Supplement: Supplementary file 3 [file Image_3.jpeg]
