# Supplementary material for: Validation and analysis of expression, prognosis and immune infiltration of WNT gene family in non-small cell lung cancer
Source: Front Oncol. 2022 Jul 25;12:911316. doi: 10.3389/fonc.2022.911316 (PMC9359207; doi:10.3389/fonc.2022.911316)
Supplement: Supplementary file 4 [file Table_1.docx]

**Table S1**. The sequences of primers used for qRT-PCR.

| **Gene Name** | **Primer Sequences (5’-3’)** | **Annealing temperature** |
| --- | --- | --- |
| *WNT7A* | F: GGCTACGTGCTCAAGGACAAGTAC | 62℃ |
|  | R: GCGGTACGACAGTGGCTTCTTG |  |
| *WNT2B* | F: TTTCTGAAGCTGGAGTGTAAGT | 62℃ |
|  | R: AAAGTAGACAAGATCAGTCCGG |  |
| *GAPDH* | F: CAGGAGGCATTGCTGATGAT | 60℃ |
|  | R: GAAGGCTGGGGCTCATTT |  |

Note: F is forward primer; R is reverse primer.
