# Supplementary material for: Validation and analysis of expression, prognosis and immune infiltration of WNT gene family in non-small cell lung cancer
Source: Front Oncol. 2022 Jul 25;12:911316. doi: 10.3389/fonc.2022.911316 (PMC9359207; doi:10.3389/fonc.2022.911316)
Supplement: Supplementary file 5 [file Table_2.docx]

**Table S2**. The clinical information of LUAD and LUSC patients.

|  | **characteristic** | **LUAD** | **LUSC** |
| --- | --- | --- | --- |
| status | Alive | 329 | 285 |
|  | Dead | 187 | 216 |
| Age | Mean (SD) | 65.3 (10) | 67.2 (8.6) |
|  | Median [MIN, MAX] | 66 [33,88] | 68 [39,90] |
| Gender | FEMALE | 278 | 130 |
|  | MALE | 238 | 371 |
| Race | AMERICAN INDIAN | 1 |  |
|  | ASIAN | 8 | 9 |
|  | BLACK | 52 | 30 |
|  | WHITE | 389 | 349 |
| pT_stage | T1 | 67 | 50 |
|  | T1a | 47 | 24 |
|  | T1b | 55 | 40 |
|  | T2 | 169 | 172 |
|  | T2a | 82 | 87 |
|  | T2b | 27 | 34 |
|  | T3 | 47 | 71 |
|  | T4 | 19 | 23 |
|  | TX | 3 |  |
| pN_stage | N0 | 332 | 319 |
|  | N1 | 96 | 131 |
|  | N2 | 74 | 40 |
|  | N3 | 2 | 5 |
|  | NX | 11 | 6 |
| pM_stage | M0 | 347 | 411 |
|  | M1 | 18 | 5 |
|  | M1a | 2 | 1 |
|  | M1b | 5 | 1 |
|  | MX | 140 | 79 |
| pTNM_stage | I | 5 | 3 |
|  | IA | 131 | 90 |
|  | IB | 140 | 151 |
|  | II | 1 | 3 |
|  | IIA | 50 | 65 |
|  | IIB | 71 | 94 |
|  | IIIA | 73 | 63 |
|  | IIIB | 11 | 18 |
|  | IV | 26 | 7 |
|  | III |  | 3 |
| new tumor event type | Metastasis | 61 | 35 |
|  | Metastasis:Primary | 1 | 2 |
|  | Metastasis:Recurrence | 8 | 3 |
|  | Primary | 11 | 12 |
|  | Recurrence | 48 | 29 |
| Smoking | Non-smoking | 75 | 18 |
|  | Smoking | 427 | 471 |
| Radiation_therapy | Non-radiation | 142 | 139 |
|  | Radiation | 13 | 15 |
